# Photogalvanic Effect in Nitrogen-Doped Monolayer MoS_2_ from First Principles

**DOI:** 10.1186/s11671-019-3222-5

**Published:** 2019-12-16

**Authors:** Wen-Ming Luo, Zhi-Gang Shao, Mou Yang

**Affiliations:** Guangdong Provincial Key Laboratory of Quantum Engineering and Quantum Materials, GPETR Center for Quantum Precision Measurement, Guangdong Engineering Technology Research Center of Efficient Green Energy and Environment Protection Materials, SPTE, South China Normal University, Guangzhou, 510006 China

**Keywords:** Photogalvanic effect, N-doped monolayer MoS_2_, Asymmetry of spatial inversion, Joint density of states

## Abstract

We investigate the photogalvanic effect in nitrogen-doped monolayer molybdenum disulfide (MoS_2_) under the perpendicular irradiation, using first-principles calculations combined with non-equilibrium Green function formalism. We provide a detailed analysis on the behavior of photoresponse based on the band structure and in particular the joint density of states. We thereby identify different mechanisms leading to the existence of zero points, where the photocurrent vanishes. In particular, while the zero point in the linear photovoltaic effect is due to forbidden transition, their appearance in the circular photovoltaic effect results from the identical intensity splitting of the valance band and the conduction band in the presence of Rashba and Dresslhaus spin-orbit coupling. Furthermore, our results reveal a strong circular photogalvanic effect of nitrogen-doped monolayer MoS_2_, which is two orders of magnitude larger than that induced by the linearly polarized light.

## Introduction

Searching for novel materials and exploring their exotic properties constitute a major theme in modern physics. At present, there exist significant interests in monolayer molybdenum disulfide (MoS_2_), which, similar as graphene, can be exfoliated mechanically [1, 2]. In contrast to the bulk MoS_2_ that belongs to an indirect-band-gap semiconductor, the monolayer MoS_2_ is a direct-band-gap semiconductor [3] with a large band gap. The monolayer MoS_2_ possesses excellent optical and electrical properties [4], such as strong photoabsorption [5–8] and high carrier mobility, which promise important applications in transistors [9] and ultra sensitive photodetectors [10]. Further, recent ab initio studies have demonstrated the possibility to tailor the electronic and magnetic properties [11–19] of monolayer MoS_2_ by doping, paving the way for spintronic devices with large latent capacity [20].

The photogalvanic effect (PGE), where electronic current is induced when the material is illuminated by light, can occur in a semiconductor with broken space inversion symmetry. The PGE can be induced by either the circularly or linearly polaried lights, which are coined as, respectively, the circular photogalvanic effect (CPGE) and linear photovoltaic effect (LPGE). Recently, the PGE has been observed in several new materials [21–26]. For example, GaAs/AlGaAs (a kind of two-dimensional electron gas) is found to exhibit both the LPGE and CPGE [27]. The CPGE has also been found in topological insulators [28–30], such as HgTe quantum wells and Sb_2_Te_3_. Remarkably, the CPGE has been reported in some Weyl semimetals [31–33]. In addition, photoresponse in graphene PN junctions and in S-doped monolayer black phosphorus [34–36] have been analysed by the team of Guo. Interestingly, both the LPGE and CPGE can exhibit zero points, where the photocurrent vanishes. However, it remains an open question as to the mechanism leading to these zero points.

Doping in monolayer MoS_2_ has been analysed by experiment [37–40] and theory [11, 41, 42], especially for nitrogen-doped monolayer MoS_2_ [38, 43]. In this work, we carry out a first-principles study of the PGE in nitrogen-doped monolayer MoS_2_. We find the material exhibits both CPGE and LPGE, which are spatially anisotropic and exhibit zero points. With a combined analysis of joint density of states (JDOS) and the band structure, we provide a detailed investigation on the behavior of photocurrent. In particular, we find that the zero points in the LPGE and CPGE arise from different mechanisms: the former is caused by forbidden transition in the former, whereas the latter is due to zero total spin slitting in presence of Rashba and Dresslhaus spin-orbit coupling.

## Model and Methods

First, the geometry optimum is performed in CASTEP Package [44, 45]. For the unit cell of nitrogen-doped monolayer MoS_2_, the generalized gradient approximation (GGA) and Perdew-Burke-Ernzerhof (PBE) parametrization were employed for the exchange and correlation potentials. To obtain a structure with high precision, the energy cutoff of plane waves was taken as 500 eV. In the reciprocal space, 6×12×1 k-points were considered. The total energy is converged to 10^−6^*eV* and the residual forces on each atom are less than 0.01 $eV/\mathop A\limits ^ \circ $.

Next, the quantum transport package *Nanodcal* [46, 47] was used for a self-consistent calculation of JDOS and the band structure, which is complemented with *GGA*_*PBE*96 for the exchange correlation functional. Here, a double zeta polarized (DZP) atomic orbital basis was used to expand all the physical quantities. Finally, the photocurrent of the device was computed within the Green’s function formalism and the density functional theory (NEGF-DFT).

**Fig. 1:**
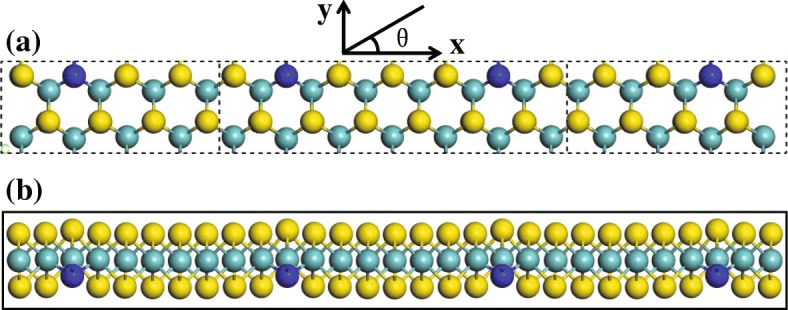
**a** Two-probe device structure for calculating the photocurrent of the nitrogen-doped MoS_2_. **b** The side view of the relaxed configuration of **a**. The S, Mo, and N atoms are respectively depicted by yellow, light blue, and dark blue. Without bias voltages, the scattering region is irradiated by the polarized light perpendicularly. For the linearly polarized light, the polarization angle *θ* is measured with respect to the transport direction

The architecture of the two-probe device is illustrated in Fig. 1. There, the sulfur atoms are doped with nitrogen-atoms, their ratio being 16:1, resulting in broken space reversal symmetry. Figure 1a shows a device exhibiting a mirror symmetry, which contains 39 atoms in the scattering region. Its side view [see Fig. 1b], a relaxed configuration obtained after structure optimization, illustrates the sandwich structure of nitrogen-doped monolayer MoS_2_.

Atoms in the scattering region of nitrogen-doped monolayer MoS_2_ were irradiated perpendicularly by light, which polarization vector can be generically described by

1 $$ \begin{array}{l} \bf{e} = \left[ {\cos \theta \cos \phi - i\sin \theta \sin \phi} \right]\mathbf{e}_{1}\\ \begin{array}{*{20}{c}} {}&{} \end{array} + \left[ {\sin \theta \cos \phi + i\cos \theta \sin \phi} \right]\mathbf{e}_{2} \end{array}.  $$

Here, *θ* labels the polarization angle of linearly polarized light, *ϕ* is the phase angle describing helicity of elliptical polarized light, and **e**_*α*_(*α*=1,2) denote unit vectors. Note that *ϕ*=±45^∘^ corresponds to the right/left-handed circularly polarized light, while *ϕ*=0 corresponds to the linearly polarized light. Since the space reversal symmetry is broken in the considered sample, PGE can be generated. Denoting the current from one lead to the center region by 〈*I*〉^(*ph*)^, we calculate 〈*I*〉^(*ph*)^ using NEGF-DFT with the quantum transport package *Nanodcal* [46, 47]. The corresponding normalized photocurrent is given by

2 $$ {R_{I}} \equiv \frac{{{{\left\langle I \right\rangle }^{\left({ph} \right)}}}}{{e{I_{\omega} }}}.  $$

Here, *I*_*ω*_ is the number of photons per unit time through per unit area, i.e., the photon flux [see Refs. [34–36, 48]]. In *Nanodcal*, the photocurrent $I_{L}^{(ph)}$ of left electrode can be given by [34]

3 $$ {{}\begin{aligned} I_{L}^{(ph)} = \frac{{ie}}{h}\int {Tr\left\{ {{\Gamma_{L}}\left[ {{G^{< \left({ph} \right)}} + {f_{L}}\left(E \right)\left({{G^{> \left({ph} \right)}} - {G^{< \left({ph} \right)}}} \right)} \right]} \right\}} dE, \end{aligned}}  $$

where *G*^<(*ph*)^ and *G*^>(*ph*)^ is lesser Green’s function and greater Green’s function respectively (with electron −photon interactions). *Γ*_*L*_ denotes the coupling of the scattering region with the left electrode. For linearly polarized light, photocurrent can be given by

4 $$ {{}\begin{aligned} \begin{array}{l} I_{L}^{(ph)}=\frac{{ie}}{h}{\int}\{{{\cos}^{2}}\theta\mathrm{{\textstyle Tr}}\left\{ {{\Gamma_{L}}\left[{G_{1}^{<\left({ph}\right)}}\right.}\right.\\ \left.\left.+{f_{L}}\left({G_{1}^{>\left({ph}\right)}-G_{1}^{<\left({ph}\right)}}\right)\right]\right\} \\ +{{\sin}^{2}}\theta\text{Tr}\left\{ {{\Gamma_{L}}\left[{G_{2}^{<\left({ph}\right)}+{f_{L}}\left({G_{2}^{>\left({ph}\right)}-G_{2}^{<\left({ph}\right)}}\right)}\right]}\right\} \\ +\sin\left({2\theta}\right){2}\text{Tr}\left\{ {{\Gamma_{L}}\left[{G_{3}^{<\left({ph}\right)}\,+\,{f_{L}}\left({G_{3}^{>\left({ph}\right)}-G_{3}^{<\left({ph}\right)}}\right)}\right]}\right\} {\left.{\vphantom{{{\cos}^{2}}\theta\mathrm{{\textstyle Tr}}}}\right\} }dE. \end{array} \end{aligned}}  $$

For circularly polarized light, it can be written as

5 $$ \begin{array}{l} I_{L}^{(ph)}=\frac{{ie}}{h}{\int}\{{{\cos}^{2}}\phi\mathrm{{\textstyle Tr}}\left\{ {{\Gamma_{L}}\left[{G_{1}^{<\left({ph}\right)}}\right.}\right.\\ \left.\left.+{f_{L}}\left({G_{1}^{>\left({ph}\right)}-G_{1}^{<\left({ph}\right)}}\right)\right]\right\} \\ +{{\sin}^{2}}\phi\text{Tr}\left\{ {{\Gamma_{L}}\left[{G_{2}^{<\left({ph}\right)}+{f_{L}}\left({G_{2}^{>\left({ph}\right)}-G_{2}^{<\left({ph}\right)}}\right)}\right]}\right\} \\ +\frac{{\sin\left({2\phi}\right)}}{2}\text{Tr}\left\{ {{\Gamma_{L}}\left[{G_{3}^{<\left({ph}\right)}\,+\,{f_{L}}\left({G_{3}^{>\left({ph}\right)}-G_{3}^{<\left({ph}\right)}}\right)}\right]}\right\} {\left.\right\} }dE. \end{array}  $$

Both of them, $G_{1}^{^{> / < \left ({ph} \right)}}$ and $G_{2}^{^{> / < \left ({ph} \right)}}$ have the same expression as followings

6 $$ G_{1}^{^{> / < \left({ph} \right)}} = \sum\limits_{\alpha,\beta = x,y,z} {{C_{0}}NG_{0}^{r}} {e_{1\alpha }}p_{\alpha}^{\dag} G_{0}^{> / < }{e_{1\beta }}{p_{\beta} }G_{0}^{a},  $$

7 $$ G_{2}^{^{> / < \left({ph} \right)}} = \sum\limits_{\alpha,\beta = x,y,z} {{C_{0}}NG_{0}^{r}} {e_{2\alpha }}p_{\alpha}^{\dag} G_{0}^{> / < }{e_{2\beta }}{p_{\beta} }G_{0}^{a},  $$

where $G_{0}^{a}$ and $G_{0}^{r}$ are the advanced and retarded Green’s functions respectively (without photons). *p*_*α*/*β*_ represents the cartesian component of the electron momentum. *e*_1/2*β*_ denotes cartesian component of the unit vector. *N* is the number of photons. ${C_{0}} = {I_{\omega } }{\left ({e/{m_{0}}} \right)^{2}}\hbar \sqrt {{\mu _{r}}{\varepsilon _{r}}} /2N\omega \varepsilon c$, where *c* is the speed and *ω* is the frequency of the light. *ε* and *ε*_*r*_ are dielectric constant and relative dielectric constant respectively. *μ*_*r*_ denotes the relative magnetic susceptibility. *m*_0_ represents the bare electron mass. For linearly polarized light,

8 $$ \begin{array}{l} G_{3}^{^{> / < \left({ph} \right)}} = \sum\limits_{\alpha,\beta = x,y,z} {{C_{0}}N\left({G_{0}^{r}{e_{1\alpha }}p_{\alpha}^{\dag} G_{0}^{> / < }{e_{2\beta }}{p_{\beta} }G_{0}^{a}}\right.} \\ \begin{array}{*{20}{c}} {}&{}&{}&{}&{}&{}&{}&{} \end{array} + G_{0}^{r}{e_{2\alpha }}p_{\alpha}^{\dag} G_{0}^{> / < }{e_{1\beta }}{p_{\beta} }G_{0}^{a}). \end{array}  $$

For circularly polarized light,

9 $$ \begin{array}{l} G_{3}^{^{> / < \left({ph} \right)}} = \pm i\sum\limits_{\alpha,\beta = x,y,z} {{C_{0}}N\left({G_{0}^{r}{e_{1\alpha }}p_{\alpha}^{\dag} G_{0}^{> / < }{e_{2\beta }}{p_{\beta} }G_{0}^{a}}\right.} \\ \begin{array}{*{20}{c}} {}&{}&{}&{}&{}&{}&{}&{} \end{array} - G_{0}^{r}{e_{2\alpha }}p_{\alpha}^{\dag} G_{0}^{> / < }{e_{1\beta }}{p_{\beta} }G_{0}^{a}). \end{array}  $$

A crucial ingredient in our subsequent analysis of PGE is JDOS, which measures the number of allowed optical transitions between the electronic states in the occupied valence band and unoccupied conduction band [49–53]. The JDOS corresponding to the excitation by photons with frequency *ω* is given by

10 $$ {J_{cv}}\left({\hbar \omega} \right) = \int\limits_{\text{BZ}} {\frac{{2d\bf k} }{{{{\left({2\pi} \right)}^{3}}}}} \delta \left[ {{E_{c}}\left(\mathbf{k} \right) - {E_{v}}\left(\mathbf{k} \right) - \hbar \omega} \right],  $$

where *E*_*c*_(**k**) and *E*_*v*_(**k**) denote the energies of electronic states at momentum **k** in the conduction and valence bands, respectively. For a two-dimensional system with nondegenerate bands, JDOS is rewritten as

11 $$ {J_{cv}}\left({\hbar \omega} \right) = \int\limits_{\text{BZ}} {\frac{{d\bf k} }{{{{\left({2\pi} \right)}^{2}}}}} \delta \left[ {{E_{c}}\left(\mathbf{k} \right) - {E_{v}}\left(\mathbf{k} \right) - \hbar \omega} \right].  $$

## Results and Discussion

**Fig. 2:**
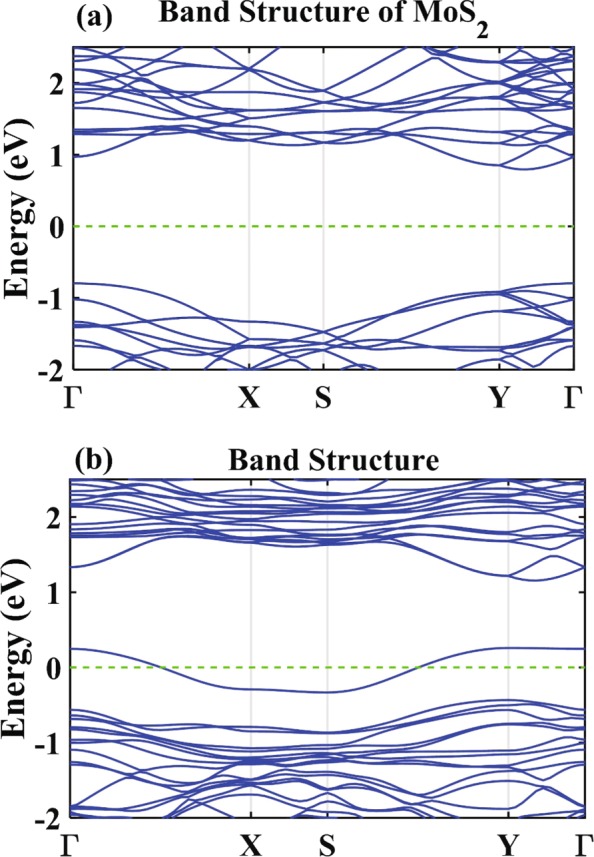
Band structure of **a** monolayer MoS_2_ and **b** nitrogen-doped monolayer MoS_2_

Figure 2 presents the band structure of monolayer MoS_2_ and nitrogen-doped monolayer MoS_2_. In the previous literatures, monolayer MoS_2_ is a direct-gap semiconductor with a band gap of 1.90 eV [3, 4]. In order to compare the band structure before [see Fig. 2a] and after doping, we select the same paths in the brillouin zone. For nitrogen-doped monolayer MoS_2_, an impurity-induced band crossing the Fermi level is observed, which is close to the top of valence bands[see Fig. 2b]. Hence, nitrogen-doped monolayer MoS_2_ is a p-type semiconductor. Importantly, because of the broken space inversion symmetry, the energy bands of the pristine monolayer MoS_2_ further split in presence of doping, even without external voltage. As is known, such splitting of energy band will allow for spin-orbit coupling under irradiation by circularly polarized light, providing an important mechanism for the CPGE.

**Fig. 3:**
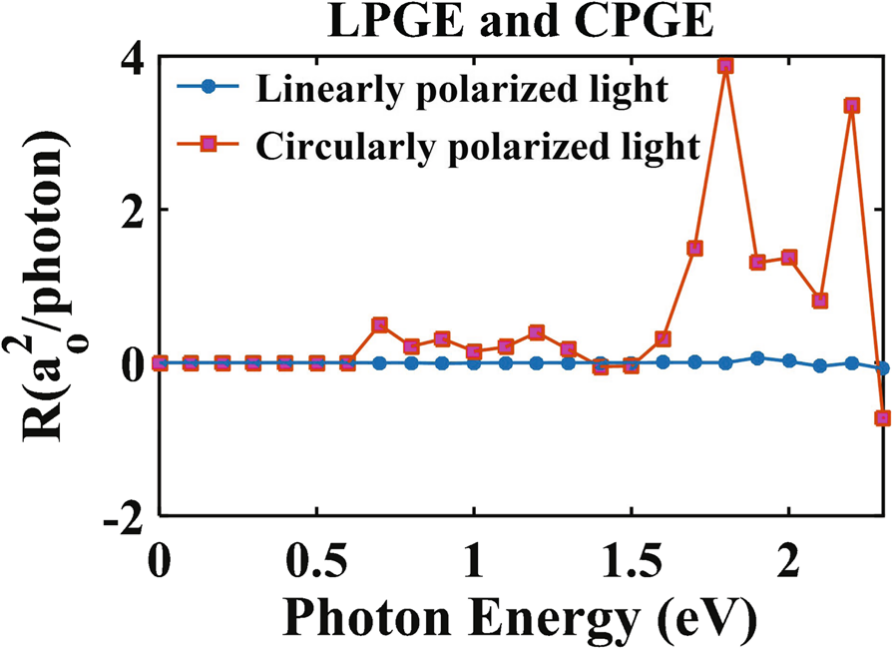
Variations of photoresponse function with the energies of the linearly polarized light and circularly polarized light, respectively. For LPGE, *θ*=*π*/4 and *ϕ*=0^∘^. For CPGE, *θ*=0^∘^ and *ϕ*=*π*/4. Photon energies range from 0 to 2.3 eV with an interval of 0.1 eV

We now study the photoresponse of nitrogen-doped monolayer MoS_2_ under perpendicular irradiation by light, obtained via NEGF-DFT calculations. Figure 3 shows the photoresponse function of LPGE and CPGE. For LPGE, *θ*=*π*/4 and *ϕ*=0^∘^. For CPGE, *θ*=0^∘^ and *ϕ*=*π*/4. The photon energy ranges from 0 to 2.3 eV (with an interval of 0.1 eV). In Fig. 3, the photoresponse of CPGE in the nitrogen-doped monolayer MoS_2_ is two orders of magnitude stronger than the LPGE. The photoresponse of LPGE stays vanishingly small in the entire regime, which is a direct consequence of the symmetry of device structure. In contrast, CPGE arises after 0.7 eV, which closes to the energy gap between impurity band to conduction band at the high symmetry point Y [see Fig. 2b]. It means that electron transition is direct. Further, CPGE becomes significant when the photon energy is above 1.7 eV. When the photon energy further increases, the photoresponse’s magnitude varies in a nonlinear fashion, while its direction switches from positive to negative.

To gain intuitions into above phenomena, we note that photocurrent is intimately connected with the photo absorption coefficient *α* defined by

12 $$\begin{array}{@{}rcl@{}} \alpha &=& \frac{{n\omega }}{{\pi}m_{e}^{2}c_{0}^{3}}\int_{\text{BZ}}d\mathbf{k}\left|\mathbf{s}\cdot\mathbf{M}_{cv}(\mathbf{k}) \right|^{2} \\ &&\delta \left[E_{c}(\mathbf{k'}) - E_{v}(\mathbf{k}) - \hbar \omega \right]. \end{array} $$

Here, *n* is the refractive index, *c*_0_ denotes the speed of light in the vacuum, and *m*_*e*_ labels the mass of electron. Further, ***s*** donotes the unit vector of the vector potential of electromagnetic wave. The matrix element **M**_*cv*_ corresponds to the momentum **p** and has the form 〈*c*,**k**|**p**|*v*,**k**〉, with |*v*(*c*),**k**〉 being the electronic state at quasimomentum **k** in the valance (conduction) band. Note that occurrence of photocurrent requires *α*>0. Equation (12) implies that the photocurrent crucially depends on both quantities: the matrix element **M**_*cv*_ and the JDOS.

**Fig. 4:**
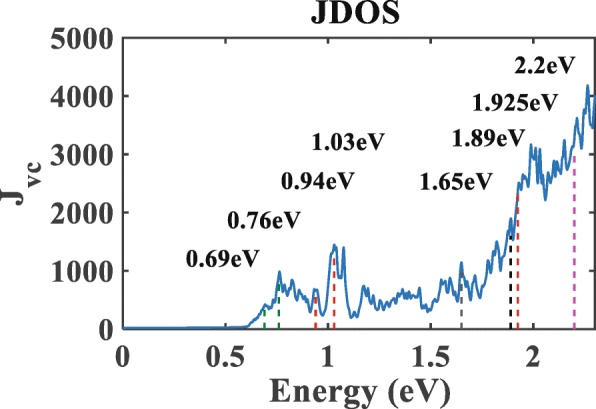
Joint density of states of nitrogen-doped monolayer MoS_2_. The dotted lines label the critical points of energies

Figure 4 shows the JDOS of the sample. JDOS nearly vanishes for photon energies below 0.5 eV, indicating electrons can hardly be excited. However, when photon energy exceeds 0.5 eV, series of peaks in JDOS arise. In Fig. 4, two peaks arise at photon energies 0.69 eV and 0.76eV (see green dotted lines). This correspond to the minimum energy to excite an electron from the valence band to the impurity band at the high symmetry point Y and *Γ* [see Fig. 2b], respectively. Furthermore, peaks are also observed when photon energies take the values of 0.94 eV, 1.03 eV, and 1.925 eV. They correspond to optical excitations of electrons from the impurity band to the conduction band at the high symmetry point Y, *Γ*, and S respectively. In addition, the peaks at 1.65 eV and 1.89 eV (black dotted lines) correspond to the electronic transition from the valence band to the conduction band at high symmetry point Y and *Γ* respectively. After 1.89 eV, JDOS increases sharply like exponential function, whose trend is in accordance with experiment of optical absorptivity [40]. Besides, our results show that nitrogen-doped monolayer MoS_2_ has a strong light absorption in the range of visible light, which is also consistent with the experimental results.

Contrasting Fig. 4 with Fig. 3 indicates an intimate connection between the JDOS and the photoresponse. There, both the JDOS and the photoresponse are nearly zero at photon energies below 0.5 eV, become nonzero—but remain small—in the regime from 0.6 to 1.7 eV, then arise significantly and fluctuate strongly in the regime from 1.7 to 2.3 eV. In particular, when the photon energies is 1.7 eV, photoresponse of CPGE exhibit pronounced peaks. Combined with Fig. 2b, we know that electrons may have two transitions using the impurity band since electrons which are excited for the photon energy of 1.7 eV from valence band to conduction are limited. However, the photoresponse owns the maximum amplitude because that the electrons can transfer from valence band to impurity band and then transfer from impurity band to conduction band.

**Fig. 5:**
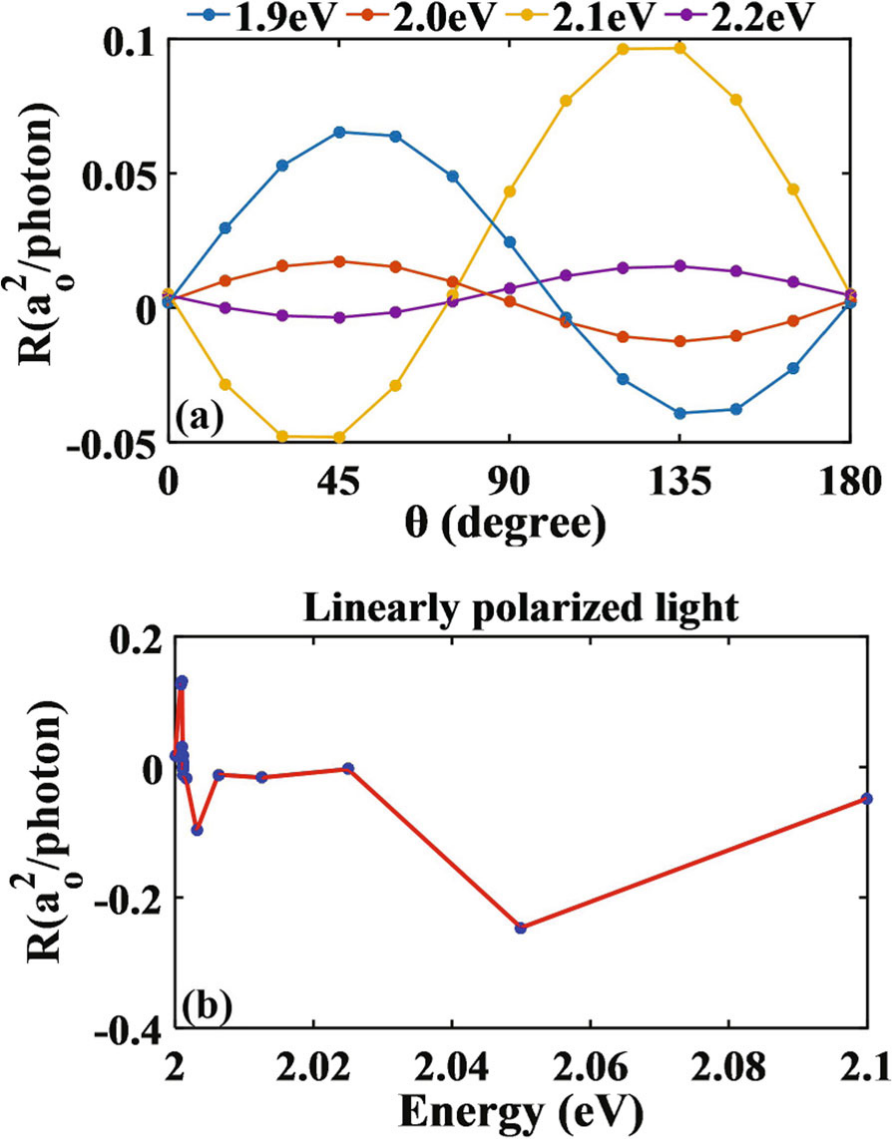
Behavior of photoresponse function for nitrogen-doped monolayer MoS_2_ irradiated by the linearly polarized light perpendicularly. Variation of photoresponse function with **a** the polarized angles and **b** the energies of linearly polarized light for *θ*=*π*/4

To further understand the behavior of photocurrent, we next plot the photoresponse of LPGE as a function of polarized angle *θ* [see Fig. 5a]. One finds the amplitude of photoresponse behaves as ∼ sin(2*θ*). This is consistent with the phenomenological theory for LPGE of a material with *C*_*s*_ symmetry under normal incidence, where ${R_{x}} {\propto } E_{0}^{2}{\chi _{xxy}}\sin \left ({2\theta } \right)$ [21, 26, 54–56] with *E*_0_ the electric field intensity of the light and *χ*_*xxy*_ being a tensor. Interestingly, while the photoresponse function behaves as sin(2*θ*) when the photon energy is 2.0 eV, it becomes instead − sin(2*θ*) when the photon energy is 2.1 eV. Therefore, there necessarily exists a point inbetween 2.0 eV and 2.1 eV where the photocurrent vanishes, i.e., the zero point of LPGE. To locate the zero point, we use a method based on dichotomy and plot the variation of photoresponse with respect to the energy of linearly polarized light. As shown in Fig. 5b for *θ*=*π*/4 degree, the zero point occurs for a photon energy of 2.0012 eV. As pointed out earlier according to Eq. (12), the photocurrent depends on both the JDOS and the matrix element of momentum. Since the JDOS is always found to be finite in our calculations, the occurrence of zero point can only be attributed to the absence of electronic transition, i.e., the existence of zero point in this case is due to the forbidden transition.

**Fig. 6:**
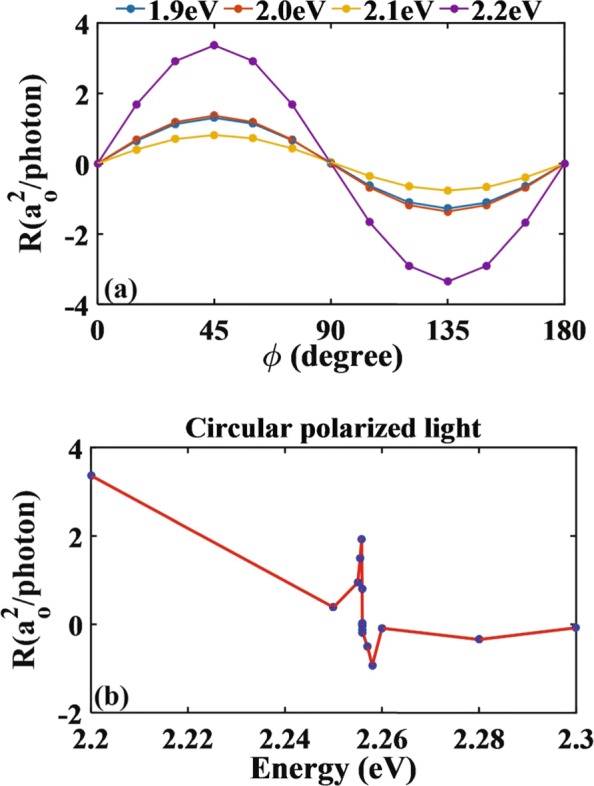
Nitrogen-doped monolayer MoS_2_ is irradiated by the elliptical polarized light perpendicularly. **a**, **b** The variations of photoresponse function with the phase angles and the energies of circularly polarized light for *ϕ*=45^*o*^, respectively

For comparison, the photoresponse of CPGE as a function of the phase angle *ϕ* is summarized in Fig. 6. One finds *R*_*x*_∼ sin(2*ϕ*), again in agreement with the phenomenological prediction which gives ${R_{x}} {\propto } E_{0}^{2}{\gamma _{xz}}\sin \left ({2\phi } \right)$ with *γ*_*xz*_ being a tensor. Similar to the LPGE, the CPGE also exhibits zero point, which occurs at 2.2560 eV in Fig. 6b. There, the transition matrix is always finite, and therefore, this zero point cannot be explained in terms of the forbidden transition as in the case of LPGE. Instead, we invoke the fact that the CPGE is deeply connected with both Rashba SOC and Dresslhaus SOC, which respectively influence the splitting of the valance band and the conduction band with different intensities. In the particular case where the splittings in the two band are identical, the excited electrons in the conduction band will have opposite momenta at ±*k*_*x*_. As a result, the net electronic current in the conduction band is zero, hence explaining the existence of zero point for CPGE.

Intriguingly, the photoresponse of CPGE in the nitrogen-doped monolayer MoS_2_ is two orders of magnitude stronger than the LPGE as shown in Figs. 3, 5, and 6. This can be understood as follows. For the LPGE, the photocurrent is induced by the asymmetric scattering of carriers. In contrast, CPGE arises because electrons in the conduction band exhibit unbalanced occupations under Rashba SOC and Dresslhaus SOC when the material is subjected to irradiation: before the illumination, due to the broken space inverse symmetry in nitrogen-doped monolayer MoS_2_, the degeneracies in the energy band of the pristine sample are lifted with Dresslhaus SOC. Then, when the material is subjected to irradiations by the circularly polarized light, the angular momentum of photons are transferred to the spin angular momentum of electrons with Rashba SOC. As an overall result, electrons fulfilling the optical-selection rule $\Delta {m_{s}} =0,\begin {array}{*{20}{c}}\end {array} \pm 1$ can be excited to the conduction band. This is different from LPGE, where the spin angular momentum of the electron remains invariant under linearly polaried light, i.e., *Δm*_*s*_=0 for LPGE. Thus, because of Rashba SOC and $\Delta {m_{s}} \begin {array}{*{20}{c}}\end {array} \pm 1$ for CPGE, the transition probability of electrons will increase dramatically for CPGE, contributing to stronger photoresponse.

Finally, seen from our calculations, photon energy is converted into electricity in our system without external bias, and the absorption of visible light is strong for nitrogen-doped monolayer MoS_2_, especially from 1.6 to 2.3 eV [see Fig. 3], i.e., from red light to green light. Therefore, it is a suitable material for 2D photovoltaic devices [57], rgb 1.00,0.00,0.00lasers [58], and single photon emitters [59]. Besides, photoresponse changes regularly with polarization and phase angles for a given photon energy as *R*∼ sin(2*θ*). Therefore, it is useful to control polarization and phase angles to dominate photocurrent. However, LPGE is slight, which prompts our experimentalists to use circularly polarized light in order to gain large photocurrent. Additionally, the analysis of JDOS with band structure gives a reasonable explanation for photocurrent, which provides a theoretical basis for the optoelectronic experimental findings.

## Conclusions

In summary, we have presented a first-principles study of PGE of in nitrogen-doped monolayer MoS_2_ under the perpendicular irradiation based on NEGF-DFT. We provide a satisfactory explanation on the behavior of photoresponse, which is achieved using a combination of analysis on the band structure and joint density of states. We find that there exist zero points in the photocurrent for both LPGE and CPGE, but the underlying mechanisms are different. For LPGE, the zero point occurs at the photon energy of 2.0012 eV, where the transition matrix element associated with electronic excitation from the valence band to the conduction band vanishes, i.e., the forbidden transition. For CPGE, on the other hand, the photocurrent is zero at the photon energy of 2.2560 eV, where, while relevant transitions are always allowed, the presence of both Rashba SOC and Dresslhaus SOC result in a net zero current. Further, the photoresponse of CPGE in the nitrogen-doped monolayer MoS_2_ is two orders of magnitude stronger than the LPGE. In general, we can change the photon energy, the type of polarized light, and the polarization angle to control the photocurrent in 2D photovoltaic devices effectively. The present theoretical work may shed light on the ongoing explorations of photogalvanic effect of nano-materials and can open up a new avenue towards optoelectronic and photovoltaic applications involving monolayer MoS_2_.

## Data Availability

All data generated or analyzed during this study are included within the article.

## Abbreviations

CPGE: Circular photogalvanic effect
LPGE: Linear photovoltaic effect
JDOS: Joint density of states
GGA: Generalized gradient approximation
PBE: Perdew-Burke-Ernzerhof
DZP: Double zeta polarized
DFT: Density functional theory
NEGF: Non-equilibrium greens function method
